# AXL is an oncotarget in human colorectal cancer

**DOI:** 10.18632/oncotarget.3962

**Published:** 2015-04-29

**Authors:** Erika Martinelli, Giulia Martini, Claudia Cardone, Teresa Troiani, Giuseppina Liguori, Donata Vitagliano, Stefania Napolitano, Floriana Morgillo, Barbara Rinaldi, Rosa Marina Melillo, Federica Liotti, Anna Nappi, Roberto Bianco, Liberato Berrino, Loreta Pia Ciuffreda, Davide Ciardiello, Vincenzo Iaffaioli, Gerardo Botti, Fiorella Ferraiolo, Fortunato Ciardiello

**Affiliations:** ^1^ Oncologia Medica, Dipartimento di Internistica Clinica e Sperimentale “F. Magrassi “, Seconda Università degli Studi di Napoli, Via S. Pansini, Napoli, Italia; ^2^ Dipartimento di Patologia Diagnostica e di Laboratorio, Istituto Nazionale Tumori- IRCCS “Fondazione G. Pascale”, Via M Semmola, Napoli, Italia; ^3^ Sezione di Farmacologia, Dipartimento di Medicina Sperimentale, Seconda Università degli Studi di Napoli, Via L. De Crecchio, Napoli, Italia; ^4^ Dipartimento di Medicina Molecolare e Biotecnologie Mediche/Istituto di Endocrinologia ed Oncologia Sperimentale del CNR ”G. Salvatore”, via Pansini, Napoli, Italia; ^5^ Oncologia Medica A, Istituto Nazionale Tumori- IRCCS “Fondazione G. Pascale”, Via M Semmola, Napoli, Italia; ^6^ Oncologia Medica, Dipartimento di Medicina Clinica e Chirurgica, Università Federico II di Napoli, Italia

**Keywords:** AXL, GAS6, colorectal cancer, foretinib, FISH

## Abstract

AXL is a tyrosine kinase receptor activated by GAS6 and regulates cancer cell proliferation migration and angiogenesis. We studied AXL as new therapeutic target in colorectal cancer (CRC). Expression and activation of AXL and GAS6 were evaluated in a panel of human CRC cell lines. AXL gene silencing or pharmacologic inhibition with foretinib suppressed proliferation, migration and survival in CRC cells. In an orthotopic colon model of human HCT116 CRC cells overexpressing AXL, foretinib treatment caused significant inhibition of tumour growth and peritoneal metastatic spreading. AXL and GAS6 overexpression by immunohistochemistry (IHC) were found in 76,7% and 73.5%, respectively, of 223 human CRC specimens, correlating with less differentiated histological grading. GAS6 overexpression was associated with nodes involvement and tumour stage. AXL gene was found amplified by Fluorescence in situ hybridization (FISH) in 8/146 cases (5,4%) of CRC samples.

Taken together, AXL inhibition could represent a novel therapeutic approach in CRC.

## INTRODUCTION

Colorectal cancer (CRC) is a prominent global health problem, representing the third most common cancer worldwide [1]. Metastatic CRC (mCRC) occurs in about 25% of patients at diagnosis [2] and it is a not curable disease in approximately 25-30% of patients, following treatment of locoregional disease; several chemotherapeutics agents including irinotecan, oxaliplatin and more recently targeted monoclonal agents, such as cetuximab, panitumumab, bevacizumab and regorafenib contributed to extend the survival of these patients [3-5]. However, resistance to both chemotherapy and molecular targeted agents might occur [6]. Identification of resistance mechanisms and activation of alternative pathways are crucial for improving therapeutic efficacy.

AXL is a member of the TAM (TYRO3, AXL, MER) receptor tyrosine kinase (RTK) family [7, 8]. The primary ligand for TAM receptors is Growth Arrest-Specific 6 (GAS6), a fairly large (75 kDa), vitamin K–dependent protein, known to activate downstream signalling [9]. Binding of GAS6 to the extracellular domain of AXL leads to dimerization of GAS6–AXL complexes. The latter results in autophosphorylation of tyrosine residues (Tyr779, Tyr821 and Tyr866) in the intracellular tyrosine kinase domain [9].

AXL is overexpressed in several human cancers, including various leukemias and solid tumours, where it is associated with a poor prognosis [10-17]. For these reasons, we examined the potential biological relevance of AXL pathway activation and its inhibition in CRC preclinical models and AXL and GAS6 expression in human CRC specimens.

## RESULTS

### Expression and activation of AXL in human colorectal cancer cell lines

**Figure 1:**
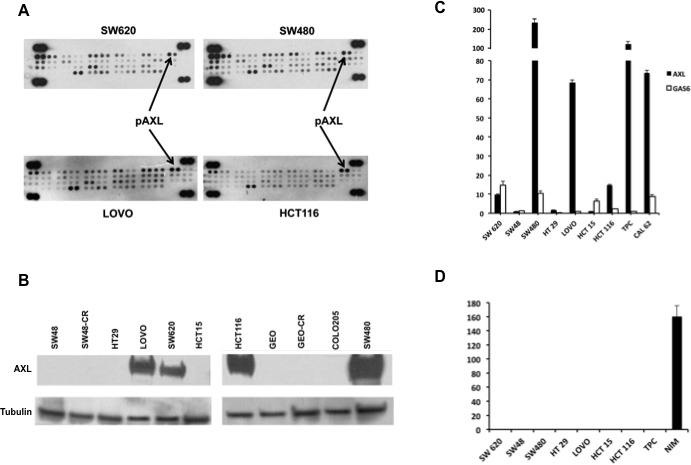
Expression and activation of AXL in human CRC cell lines **A.** 300 μg of protein lysates were obtained from human CRC cell lines SW620, SW480, LOVO, HCT116 and were analysed by human phospho-kinase array evaluating the following receptors: AXL, EphA1, EphA2, EphA3, EphA6, EphA4, EphA7, EphB1 EphB2, EphB4, EphB6, ErbB2, ErbB3, ErbB4, EGFR, FGF R1, FGFR2a, FGF R3, FGFR4, Flt 3, HGF R, insulinR, IGF-I R, Mer, MSPR, MCSFR, MuSK, PDGFrα, PDGrβ, SCFR, cRET, ROR1, ROR2, Tie2, TrkA, TrkB, TrkC, VEGFR1, VEGFR2, VEGFR3, Tie1. **B.** Western blot analysis of AXL receptor in SW48, SW48-CR, HT29, LOVO, SW620, HCT15, HCT116, GEO, GEO-CR, COLO205, SW480. Thirty micrograms of cell protein extracts were fractionated through 4% to 20% SDS-PAGE, transferred to nitrocellulose filters, and incubated with the indicated antibodies as described in Materials and Methods. Immunoreactive proteins were visualized by enhanced chemiluminescence. **C.** specific mRNA expression by quantitative real time-PCR: total RNA was extracted from colorectal (SW620, SW48, SW480, HT29, LOVO, HCT15, HCT116) and thyroid cancer cell lines (TPC, CAL 62) quantitative real time-PCR was done to assess the expression of AXL and GAS6 mRNA. **D.** GAS6 protein levels were measured in cell culture media of CRC cancer (SW620, SW48, SW480, HT29, LOVO, HCT15, HCT116) and thyroid (TPC, NIM) cancer cell lines by using specific ELISA as described in Materials and Methods.

We first performed a screening with a receptor tyrosine kinase array in four human CRC cell lines (SW620, SW480, LOVO, HCT116) non-responsive to the anti-EGFR targeting drugs, as they harbour mutations in *KRAS* genes [18-22]. Among several receptors involved in CRC tumorigenesis, AXL phospho-protein was found in all four human CRC cell lines (Figure 1A). As illustrated in Figure 1B, AXL protein expression was also confirmed in SW620, SW480, LOVO, HCT116, whereas no expression was found in the remaining cells: SW48, HT29, HCT15, GEO, COLO205, GEO-CR and SW48-CR, these two latter cell lines present acquired resistance to cetuximab [23]. *AXL* and its ligand *GAS6* mRNA were also screened by real time-PCR (RT-PCR) using TPC1 and CAL62, two thyroid cancer cell lines, as positive controls. *AXL* mRNA was detected at variable levels ranging between 1 and 238,8 fold as compared with TPC1 and CAL62 in the cell lines tested, being barely detected in SW48, HT129 and HCT15. *GAS6* mRNA was weakly found in all CRC cell lines (range 1-14,9) (Figure 1C).

We analysed the secretion of GAS6 into the cell culture media (CM). Forty-eight hours (hrs) after cell seeding, cancer cells were serum starved and collected after additional 24 hrs. As shown in Figure 1D, GAS6 was secreted only by thyroid cancer cells (NIM) that were used as a positive control (Figure 1D), suggesting a ligand-independent activation of AXL in human CRC cells.

In order to identify genes or pathways related with AXL expression, we analysed baseline microarray gene expression of CRC cell lines expressing AXL compared to CRC cell lines defined as AXL negative. In this respect, we found 1553 and 1061 genes defined as up-regulated or down-regulated, respectively, in AXL positive cancer cell lines (t test, *p* < 0.05) (data not shown). Among the up-regulated genes, 33 genes are involved in epithelial to mesenchymal transition (EMT) (Table 1).

**Table 1:** Common up-regulated genes in AXL expressing CRC cell lines

GENE SYMBOL	GENE NAME	FOLD CHANGE
ADAM11	ADAM metallopeptidase domain 11	10,7943
AHNAK2	AHNAK nucleoprotein 2	7,985296
AKT3	v-akt murine thymoma viral oncogene homolog 3	12,12195
BCL2	B-cell CLL/lymphoma 2	9,289825
BMP6	bone morphogenetic protein 6	54,55846
BMP7	bone morphogenetic protein 7	66,8874
BMP8A	bone morphogenetic protein 8a	26,31088
BMP8B	bone morphogenetic protein 8b	8,796837
CALD1	caldesmon 1	103,4702
CAV2	caveolin 2	4,0217
COL3A1	collagen, type III, alpha 1	16,79685
FLT1	fms-related tyrosine kinase 1	11,51779
FN1	fibronectin 1	18,60154
GLI1	GLI family zinc finger 1	8,795941
GLI2	GLI family zinc finger 2	9,151444
GLI3	GLI family zinc finger 3	7,520125
MITF	microphthalmia-associated transcription factor	11,63769
MSN	moesin	92,42856
NRG1	neuregulin 1	25,02382
SMO	smoothened, frizzled family receptor	94,62124
SNAI3	snail homolog 3 (Drosophila)	4,282289
SPARC	secreted protein, acidic, cysteine-rich (osteonectin)	48,42324
TCF4	transcription factor 4	12,59067
TGFB1	transforming growth factor, beta 1	5,194356
TGFB1I1	transforming growth factor beta 1 induced transcript 1	4,151411
TGFB2	transforming growth factor, beta 2	8,969274
TMEFF1	transmembrane protein with EGF-like two follistatin-like domains 1	4,351279
TMEM132A	transmembrane protein 132A	4,58216
TWIST1	twist homolog 1 (Drosophila)	6,788473
VCAN	versican	10,09725
VIM	vimentin	13,15245
WNT5A	wingless-type MMTV integration site family, member 5A	25,32227
ZEB1	zinc finger E-box binding homeobox 1	4,082564

Microarray gene expression analysis was performed in CRC cell lines expressing AXL and CRC cell lines defined as AXL negative. Using the Student's t test with Benjamini-Hochberg multiple test correction, 1553 and 1061 genes were identified as up-regulated or down-regulated, respectively, in AXL positive CRC cells (t test, *P* < 0,05). Among the up-regulated genes we identify 33 genes involved in epithelial to mesenchymal transition (EMT) listed in the table.

### AXL blockade inhibits colorectal cancer cell proliferation and survival

**Figure 2:**
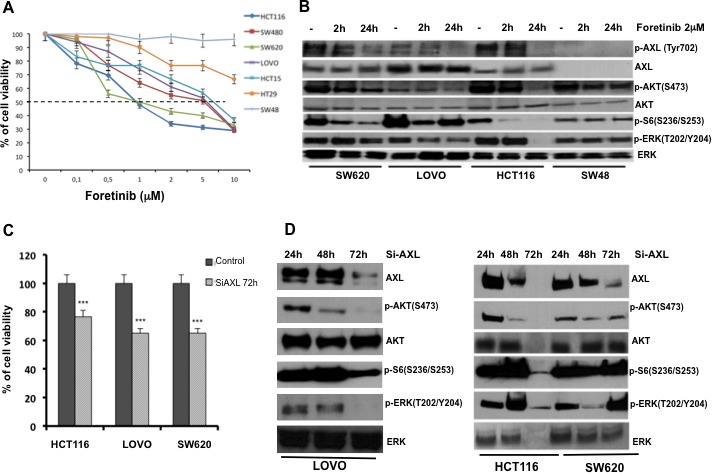
Effects of AXL blockade on CRC cancer cell proliferation and survival **A.** Effects of foretinib on cell proliferation in the panel of human CRC cell lines. As described in Materials and Methods, cancer cells were treated for 72 hrs with increasing concentration of foretinib (0.01–10 μM) and evaluated for proliferation by MTT staining. The results are average ± SD of three independent experiments each done in triplicate. **B.** Analysis of intracellular signalling pathways by Western blot in SW620, LOVO, HCT116, SW48 cancer cells treated with foretinib at indicated doses for 2 and 24 hrs. Total cell protein extracts (30 μg) were subjected to immunoblotting with the indicated antibodies as described in Materials and Methods. **C.** HCT116, LOVO and SW620 cancer cell lines were transfected with 100 nmol/L *AXL* siRNA for 72h evaluated for proliferation by MTT staining. **D.** 30 μg of protein lysates derived from HCT116, LOVO and SW620 *AXL* siRNA were extracted and analysis of intracellular signalling pathways with the indicated antibodies was performed by Western blot as described in Materials and Methods.

Inhibition of AXL might be a novel therapeutic approach to treat CRC. Therefore, we evaluated the effects on cell growth proliferation of foretinib, which is an oral multikinase inhibitor of c-MET and VEGFR2 and that was recently described as a potent inhibitor of AXL [24]. We tested treatment with foretinib in HCT116, SW480, SW620, LOVO, HCT15, HT29 and SW48. Cancer cells were treated with foretinib at dose concentrations ranging from 0.1 to 10 μM for 72 hr. The drug concentrations required to inhibit cell growth by 50% (IC_50_) were determined by interpolation from the dose-response curves. As shown in Figure 2A IC_50_ values ranging between 1 μM and > 5 μM. The most sensitive cells to foretinib were HCT116 and SW620 (IC_50_1μM) whereas the most resistant were HT29 and SW48 cells with little or no growth inhibition even up to 10μM concentration of the drug.

To determine the inhibition of intracellular signals for cell survival and proliferation, Western blot analysis were performed on protein extracts derived from SW620, LOVO, HCT116 and SW48 cancer cells, treated with foretinib (2 μM) for 2 and 24 hrs. Foretinib treatment decreased the levels of active phosphorylated AXL (pAXL) and its downstream pathway in SW620, LOVO and HCT116 cells (Figure 2B).

**Figure 3:**
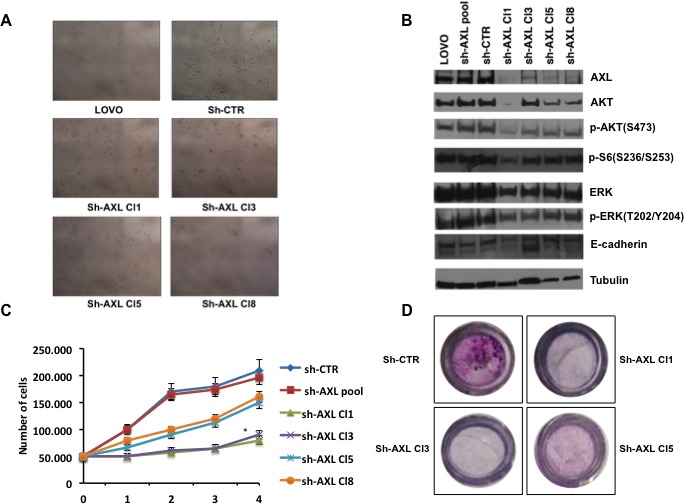
Effects of AXL gene stable silencing in LOVO CRC cells **A.** Stable transfected LOVO CRC cells with a pool of vectors expressing 5 different shRNAs directed against *AXL* or expressing control non targeting shRNAs. Four sh*AXL* clones were identified. **B.** Analysis of intracellular signalling pathways by Western blot in sh*AXL* clones (Cl1,3,8). Total cell protein extracts (30 μg) were subjected to immunoblotting with the indicated antibodies as described in Materials and Methods. **C.** cell growth curves of sh*AXL* clones; 50.000 cells of sh-CTR, sh-*AXL* pool, sh*AXL* 1,3,5,8 clones were seeded on day 0 and the total number of cells was counted every day until day 4. Every day detached cells were stained with Trypan blue 0,4%, and nonviable blue-stained cells were counted. Each point represents the mean value of 3 culture dishes, *p < 0.05. **D.** migration assay of sh*AXL* CRT,1,3,5 clones sh*AXL* 1,3,5,8 after 24 hrs.

To further evaluate AXL inhibition effects, we used a RNA interference approach. LOVO, HCT116 and SW620 human CRC cells were transfected with AXL specifics siRNAs. AXL silencing was verified by Western blot. AXL knockdown correlated with a significant inhibition of proliferation evaluated by 3-(4,5-dimethylthiazol-2-yl)-2,5-diphenyltetrazolium bromide (MTT) in HCT116, LOVO and SW480 of 78% and 68%, respectively (*p* < 0.001) (Figure 3C). Moreover, seventy-two hrs after AXL silencing, a reduction of 5-bromo-2-deoxyuridine (BrdU) incorporation of 70 % was observed (data not shown). Inhibition of AXL gene expression was also accompanied by a reduction of phosphoERK (pERK), phosphoAKT (pAKT) and phospho ribosomal protein S6, 24, 48 and 72 hrs after AXL silencing, (Figure 2D).

### Biological effects of AXL gene stable inhibition in LOVO human colorectal cells

We stably transfected LOVO CRC cells with a pool of vectors expressing 5 different shRNAs directed against *AXL* or expressing control non-targeting shRNAs. Stable transfectants were selected and amplified in medium with 500ng/mL puromycin for 12 weeks. We identified clones (Figure 3A) that expressed intermediate (sh*AXL*Cl 3) or low (sh*AXL*Cl 1,5 and 8) AXL levels (Figure 3B). We tried also to establish HCT116 stable transfectants but HCT116 were not able to survive after *AXL* knockdown, suggesting AXL relevant role in HCT116 cell survival (data not shown). In sh*AXL*1,3,5,8 clones, the activation of the MAPK and PI3K pathways (pAKT and of pS6 ribosomal protein) were decreased with respect to control (sh-*AXL* pool and sh-CTR) or parental cells (Figure 3B). Furthermore, E-cadherin levels were slightly increased in shA*XL* 1,3,5,8 clones, indirectly suggesting that *AXL* is a marker of EMT (Figure 3B).

We verified whether *AXL* knockdown could inhibit cell growth. *AXL* silencing showed a statistical significant suppression of cell growth in sh-Cl1 and 3 (*p* < 0.05) (Figure 3C). We also performed the trypan blue exclusion viability assay and we verified that sh-AXL clones did not showed any trypan blue staining, suggesting a delay of cell growth rather then cell death (data not shown).

To investigate the role of AXL in CRC cell migration we used transwell cell culture chambers. Sh*AXL* clones (Cl1, 3 and 5) showed a clear decrease of invasive ability with respect to shCTR cells after 24 hrs. These data confirm that AXL is an important mediator of migration and invasion in these CRC cells (Figure 3D).

### Pharmacologic inhibition of AXL signalling activation with foretinib in a human colorectal cancer orthotopic xenograft model

We further investigated the *in vivo* antitumor activity of foretinib. We developed a colon orthotropic model of human HCT116 cells in nude mice. We did not use LOVO AXL knockdown clones for *in vivo* experiments because of their low rate of proliferation that could affect sub-cutaneous growth.

**Figure 4:**
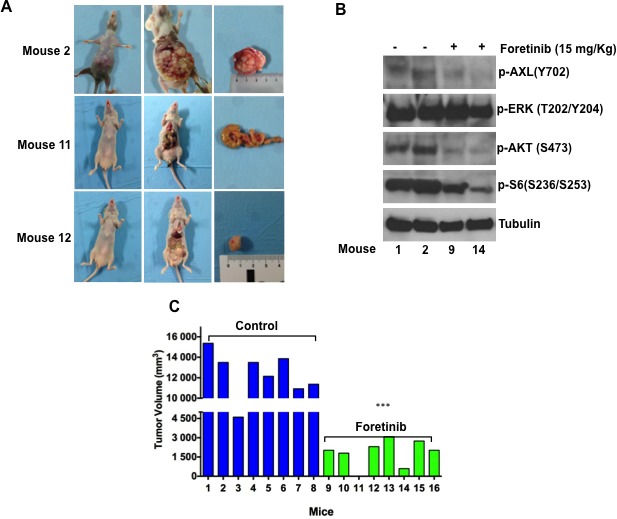
Foretinib inhibits growth of HCT116 CRC orthotopic xenograft models HCT116 subcutaneous tumour xenografts were implanted in to the cecal wall of mice. After one week mice were randomized and divided in two different groups and treated with vehicle or foretinib at the dose of 15mg/kg (oral gavage once a day) for 3 weeks (5 days a week). Animals were sacrificed 1 week after stopping treatment. **A.** Tumour incidence and presence of visceral metastasis. **B.** Western blot analysis of intracellular signalling pathways in mice treated with foretinib and in the control arm. Total cell protein extracts (30μg) were subjected to immunoblotting with the indicated antibodies as described in Materials and Methods. **C.** Tumour volume (mm^3^) of control and treated mice are compared. Each group consisted of 8 mice. ***p < 0.0001.

Briefly, a total of 2 × 10^6^ HCT116 cells re-suspended in 200 μl of matrigel and PBS (1:1) were implanted subcutaneously into the right flank of nude female mice. When the average tumours reached a mean volume of 500 mm^3^ animals were euthanized, the tumours were removed and divided into 2-3 mm-sized pieces and harvested in PBS. Mice were treated with antibiotics, ticarcillin (50 mg kg^−1^i.v.), two hrs before and after tumour implantation (for more details refer to Materials and Methods). After 10 days from the orthotopic implantation of tumour in to cecum wall of the mice, foretinib treatment was started (15mg/kg) per os 5 days a week for three weeks. As shown in Figure 4A and Table 2, the control group mice had large tumours in cecum with a 100% incidence of regional lymph nodes and peritoneal metastasis, showing, furthermore, worst clinical conditions and weight loss. In particular, in the control arm the mean weight was statistically lower compared to the treatment arm (20,5 gr versus 23,5 gr; *p* < 0.0001) (Table 2). In the foretinib treated arm, the necropsy of mice revealed no evidence of peritoneal disease and metastasis in visceral organs (intestine, rectum, liver, spleen and lung) (Figure 4A). We performed a Western blot analysis for the expression of AXL and the activated downstream pathways in the tumours: pAXL, pAKT and pS6 ribosomal protein levels were decreased in foretinib treated mice. No evident effect on ERK phosphorylation was observed (Figure 4B).

Finally, in mice treated with foretinib, tumour growth was significantly (*p* < 0 0.0001) decreased compared with the control group, with a complete response (defined as the absence of tumour at necropsy) evidenced in one mouse (Figure 4C). Foretinib treatment was well tolerated by mice, with no signs of acute or delayed toxicity.

**Table 2:** Mice tumour diameter and weight, mice weight in control and foretinib groups

Control	Tumour diameter (cm)	Tumour weight (gr)	Mouse weight (gr)
1	3,1×3,2	5,1	17
2	3×3	5,1	21,3
3	2,3×2	2,8	17,7
4	3×3	4,9	21,8
5	3,1×2,8	5	20,3
6	3,3×2,9	4,8	22
7	3×2,7	4,9	21,7
8	2,9×2,8	4,7	22
Foretinib			
9	1,8×1,5	2	17
10	1,6×1,5	3,6	22,5
11	0,2×0,2	0	26,7
12	1,8×1,6	1,2	22,3
13	1,8×1,3	1,3	24,3
14	1,2×1	0,5	29,1
15	1,9×1,7	2,1	21,3
16	1,8×1,5	2,2	23

HCT116 subcutaneous tumour xenografts were implanted in to the cecal wall of mice. After one week mice were randomized and divided in two different groups and treated with vehicle or foretinib at the dose of 15mg/kg (oral gavage once a day) for 3 weeks (5 days a week). Mice tumour diameter, tumour weight and mice weight (p<0.0001) are indicated in the table.

### Expression of AXL and GAS6 proteins in human colorectal cancer and AXL gene amplification

We evaluated whether AXL and its ligand GAS6 were expressed in CRC specimens. A total of 223 patients diagnosed with CRC were included. Baseline clinicopathological characteristics of patients are summarized in Table 3. The median follow up time was 39.5 months.

**Table 3:** Demographic distribution of patients for the total study population

Characteristics	N=223(%)
Age (yr)	
Median	59
Range	29-80
Sex	
Male	130
Female	93
Site of primary Tumor	
Right colon	97 (43,5)
Left colon or rectum	126 (56,5)
Grading	
G1	7 (3,1)
G2	179 (80,3)
G3	37 (16,6)
Tumor	
T1	10 (4,5)
T2	61 (27,4)
T3	129 (57,8)
T4	23 (10,3)
Lymph node status	
N0	93 (41,7)
N1	65 (29,1)
N2	65 (29,1)
Localized	152 (68,2)
Metastatic	71 (31,8)

Histological blocks of 223 patients diagnosed with CRC undergone to surgical resection treatment between 2003 and 2011 were selected. Patient's clinic-pathologic characteristics are listed in the table.

**Figure 5:**
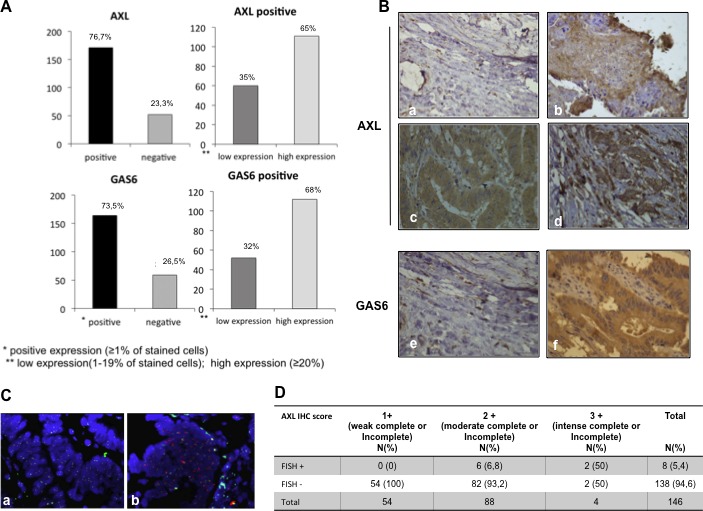
Expression of AXL and GAS6 proteins in human colorectal cancer **A.** Distribution of AXL and GAS6 expression (% of cells staining) measured by using IHC in all available CRC patients (left part). **B.** representative figures of AXL and GAS6 IHC status in the study population. Different staining intensities (40 x original magnification) in AXL negative (a), low (b), moderate (c), high (d) intense AXL positive tumour tissues and GAS6 negative (e) and positive (f) specimens. **C.** Representative figures of *AXL* gene status (red-orange signal) and CEN 19q (green signal) measured by using FISH in a small cohort of tumour cases (*N* = 146), (a) non amplified sample, AXL/CEP19 ratio:1 (b) amplified sample, harbouring *AXL* gene amplification, AXL/CEP19 ≥ 2. **D.** correlation between AXL IHC expression and FISH analysis; IHC and FISH analysis were described in Materials and Methods, ***p < 0.0001(Pearson chi-square test).

AXL protein expression by immunohistochemistry (IHC) was detected in 171 out of 223 tumour specimens (76.7%) whereas AXL expression was undetectable in 52 out of 223 (23.3%) (Figure 5A, Figure 5B,a). Among AXL positive cases, 111 (65%) showed high expression levels (staining in ≥20% cancer cells), whereas 35% showed low expression (staining in < 20% cancer cells) (Figure 5A). AXL staining mostly displayed membrane positivity; in a small number of cases cytosolic staining was observed and in one specimen nuclear staining was detected. Tumoral stroma and non-tumoral adjacent tissues were AXL negative, 10% of cases had endothelial cells positivity. Colon normal tissue did not express AXL (data not shown).

We further examined AXL intensity expression pattern. Ninety-five percent of positive specimens showed an incomplete baso-lateral membranous pattern. The remaining cases were characterized by a complete intensity pattern; 76 cases were scored as low (1+) (Figure 5B,b), 90 cases as moderate (2+) (Figure 5B,c), 5 cases as high (3+) (Figure 5B,d). A significant correlation (*p* < 0.0001) between cancer cell percentage staining and intensity expression levels was found.

Since AXL was overexpressed in approximately 2/3 of AXL positive cases, we evaluated if gene amplification could occur. Fluorescence in situ hybridization (FISH) analysis was performed in 146 AXL positive specimens. Eight cases (5.4%) showed AXL gene amplification (Figure 5C,b) with AXL/CEP19 ratio ranging from 2 to 2.5, whereas 138 cases (94.6%) were FISH negative (Figure 5C,a). A high significant correlation between AXL protein expression levels and gene amplification was observed (*p* < 0.0001). Within the 8 AXL gene amplified specimens, high intense (3+) AXL protein expression was found in 2 cases, AXL moderate intensity expression pattern (2+) was detected in the other 6 cases. No cases with AXL low intense (1+) were found FISH positive (Figure 5D).

Furthermore, we analysed GAS6 expression levels in the same specimens. We found 164 out 223 cases (73.5%) that stained positively (Figure 5A), whereas 59 cases (26.5%) were negative for GAS6 expression (Figure 5A, 5B,e). Within the positive specimens, 52 (32%) were classified as low expression (staining in < 20% cancer cell), 112 as high expression (staining in ≥20% cancer cell) (Figure 5A, bottom part).

**Figure 6:**
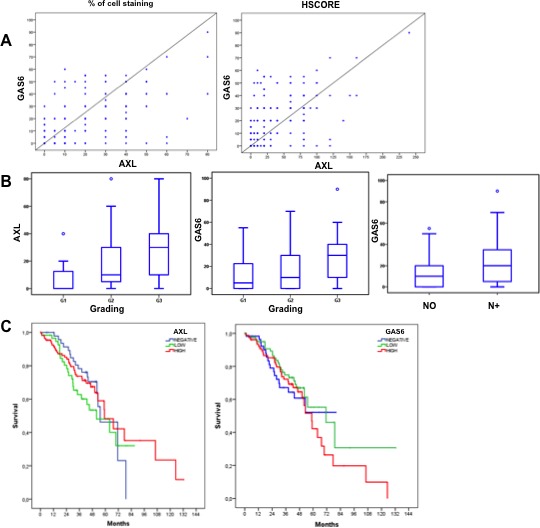
AXL and GAS6 clinicopathological features correlation and prognostic relevance **A.** correlation between AXL (% of cells staining and HSCORE) and GAS6 expression levels, by using Pearson correlation coefficient. **B.** correlation between AXL expression levels (% of cells staining) and grading and GAS6 expression levels with grading and lymph nodes status by using non-parametric Mann-Whitney U test and Kruskal-Wallis test when indicated. **C.** univariate analysis of overall survival (Kaplan-Meier method) in CRC patients data-set using the Log-rank (Mantel-Cox) statistical test stratify by AXL and GAS6 expression.

GAS6 showed a iuxtamembranous staining, 10% of cases displayed stromal positivity, indicating that the ligand can be provided by tumour microenvironment. A significant statistical correlation between AXL and GAS6 expression was found (*p* < 0.0001) (Figure 6A). To investigate the clinical relevance of AXL and GAS6 status, we evaluated the association between patients clinicopathological features and their expression levels. Neither AXL nor GAS6 were associated with age, sex, tumour primary location, tumour depth and presence of distant metastasis at initial diagnosis (*p* > 0.05; data not shown). Both AXL and GAS6 expression levels were correlated with histological grading (*p* < 0.05; Figure 6B) resulting markedly expressed in less differentiated specimens. GAS6 but not AXL was associated with lymph nodes status (*p* < 0.05; Figure 6B) and consequently with tumour stage (*p* < 0.05) showing a higher expression in more advanced disease (data not shown).

To evaluate the prognostic significance of AXL and GAS6 expression, survival analysis was performed by using Log-rank (Mantel-Cox) statistical test. In the AXL negative patients population median Overall Survival (OS) was 55.2 months (95% CI 41.5-68.9); in AXL-positive low expression patients, OS was 51.8 months (30.6-73.1) whereas in AXL-positive high expression patients, OS was 59.2 months (95% CI 43.8-74.5). AXL expression was not prognostic in our CRC dataset (*p* > 0.05; Figure 6C). In GAS6 negative patient population the median OS was 55.8 months (95% CI 46.9 - 64.7); among the GAS6-positive subgroups classified as low expression, OS was 74.6 months (95% CI 55.8 - 93.5), in GAS6-positive high expression, OS was 59.6 months (95% CI 48.0 - 71.2). GAS6 had no apparent impact on survival (Figure 6C). Similar data were obtained with HSCORE (refer to Materials and Methods for details) values (*p* > 0.05) and AXL intensity expression levels (*p* > 0.05) (data not shown).

## DISCUSSION

Colorectal cancer is a complex heterogeneous disease with defined subtypes based on different gene expression profıles. Efforts have been made to find a “consensus” subtypes classification useful to tailor therapy for CRC patients. According to gene expression profiles and genomic characterization, recently, at least three CRC subgroups have been proposed: microsatellite instability (MSI), chromosomal instability (CIN) epithelial/proliferative and CIN mesenchymal/invasive phenotype [25].

Critical genes and pathways important in the initiation and progression include the WNT, RAS, MAPK, PI3K, TGFβ, p53 and DNA mismatch-repair pathways that, when genetically altered, negatively impact on drug antitumor activity. Examples include the lack of benefıt of anti-EGFR monoclonal antibodies (moAbs) in presence of RAS mutation [22]. Moreover, recently it has been described that MET or HER2 gene amplification could be involved in primary and acquired resistance mechanisms to anti-EGFR moAbs [21, 23].

In this scenario, AXL receptor might be considered a novel potential target as its overexpression has been reported in a variety of human cancers and it has been associated with worse prognosis [10-17, 26].

To investigate the role of AXL in cancer progression we used a panel of human CRC cell lines were we detected AXL protein expression and activation. Inhibiting AXL pathway by using specific silencing RNA or a pharmacologic approach with foretinib, we demonstrated that cancer cell proliferation and migration was significantly suppressed. We further developed, in our laboratory, a model of human colon orthotopic xenograft nude mice, to evaluate the pharmacological inhibition of AXL activation. Foretinib treatment caused significant inhibition of tumour growth and peritoneal metastatic spreading compared with untreated controls confirming the *in vitro* findings.

The preclinical findings described in our manuscript demonstrated that the antitumor activity obtained by AXL inhibition is clinical relevant, because all human CRC cell lines used do not respond to the common anti-EGFR moAb due to *KRAS* mutations that confer primary resistance to these agents. In this respect, several studies suggest that overexpression of AXL may be implicated in resistance to both targeted therapies and conventional chemotherapy in different cancer models [7, 27]. In a recent study it has been reported that in cellular lung cancer models of acquired resistance to the EGFR TKI erlotinib there was an increased activation of AXL and induction of an EMT-like state [28-29]. Furthermore, Brand and colleagues demonstrated that AXL is involved in acquired resistance to cetuximab in models of head and neck squamous cell carcinoma (HNSCC), in which it was overexpressed, activated and tightly associated with EGFR expression in cancer cells resistant to cetuximab [30].

However, in the present study we did not find AXL pathway activation in two models of acquired resistance to anti-EGFR antibodies cetuximab (GEO-CR and SW48-CR) that were generated in our laboratory. As a possible explanation, in these two CRC cell lines (GEO-CR and SW48-CR) acquired resistance to cetuximab was meanly due to TGFα mediated heterodimerization of MET with EGFR [23] and by activation of angiogenesis [31].

EMT is a process in which epithelial cells exert the ability to migrate and invade surrounding tissues [32]. In this respect, in cancer cells with AXL overexpression, 33 genes involved in EMT were found up-regulated with a median fold-change of 22.7. Our findings support recent studies showing that AXL expression was associated with EMT [33-36] and in some case this phenomenon was driven by autocrine interactions with GAS6 produced by surrounding endothelial cells [10]. AXL can be activated through a number of different mechanisms: ligand-dependent dimerization (principally driven by GAS6), ligand-independent dimerization, interaction between two monomers on neighbouring cells and heteromeric dimerization with other receptors [37-38]. We did not find GAS6 secretion into cell culture media suggesting a ligand-independent activation of AXL in our *in vitro* model.

We finally determined the prevalence and clinicopathological significance of AXL and GAS6 expression in 223 human CRC tissues. In particular, we showed that AXL and GAS6 overexpression is a molecular trait of approximately 70% of CRC with a different degree of expression, evaluated by IHC, with a statistical significant correlation between the ligand and receptor. Although AXL overexpression has been previously reported in several solid tumours [10-17] this significant correlation has never been reported, suggesting the presence of an autocrine pathway between AXL and its ligand GAS6 in human CRC tissues.

AXL and GAS6 were markedly present in less differentiated tumour specimens and only GAS6 was associated with lymph nodes status and consequently with tumour stage, showing a higher expression in more advanced disease. Neither AXL nor GAS6 had any apparent impact on survival, at least in our dataset. AXL overexpression in CRC was reported in metastatic lesion [39], moreover Dunne ad colleagues found that AXL was a strong independent negative prognostic factors in early stage of CRC [40].

Notably, this is the first study reporting *AXL* gene amplification in 5,4% of CRC specimens that overexpressed AXL protein; a significant correlation between AXL protein intensity and gene amplification was found.

Different preclinical evidences define AXL a potential target to inhibit [7]. A recent report found AXL mutations in CRC cell lines never described before and many multi-kinase inhibitors blocking also AXL are under investigations with no specific indication for CRC [13].

Collectively, the results herein reported highlight AXL tyrosine kinase as a therapeutic target that is worth to be explored in a clinical setting for CRC.

## MATERIALS AND METHODS

### Drugs and chemicals

Foretinib was purchased from Selleck Chemicals (Selleckchem, Houston, TX, USA). It was dissolved in sterile Dimethylsulfoxide (DMSO) at 10 mM stock solution concentration and stored in aliquots at −20 °C. Working solutions were diluted in culture medium before each experiment and the 10 mM stock solution was stored at −20°C. For *in vivo* applications, foretinib was solubilized in 0.5% Tween-80 in sterile Phosphate Buffered Saline (PBS).

### Cell lines

Human HT29, LOVO, HCT15, HCT116, COLO205 and SW620 colorectal cancer cell lines were obtained from the American Type Culture Collection (ATTC) and have been authenticated by IRCCS “Azienda Ospedaliera Universitaria San Martino-IST Istituto Nazionale per la Ricerca sul Cancro, Genova,” Italy in 2013. The human SW48 (catalogue number: HTL99020), HCT116 (catalogue number: HTL95025), SW480 (catalogue number: HTL99017) dimethylsulfoxide cell lines were obtained from IRCCS “Azienda Ospedaliera Universitaria San Martino-IST Istituto Nazionale per la Ricerca sul Cancro, Genova,” Italy. The human GEO colon cancer cell line was kindly provided by Dr. N. Normanno (National Cancer Institute, Naples, Italy). GEO-CR and SW-48 CR cells were established as previously described (19). GEO and GEO-CR cell lines were grown in DMEM (Lonza), supplemented with 20% fetal bovine serum (FBS; Lonza) and 1% penicillin/streptomycin (Lonza). SW48, SW48-CR, LOVO, HCT15, HCT116 and SW480 cells were grown in RPMI- 1640 (Lonza) supplemented with 10% FBS and 1% penicillin/streptomycin. SW620 and HT29 cancer cells were grown in McCoy medium (Lonza) supplemented with 20% FBS (Lonza) and 1% penicillin/streptomycin (Lonza). All cell lines were grown in a humidified incubator with 5% of carbon dioxide (CO_2_) and 95% air at 37°C. All cell lines were routinely screened for the presence of mycoplasma (Mycoplasma Detection Kit; Roche Diagnostics).

### Proliferation assay

Cell proliferation was analysed by the 3-(4,5-dimethylthiazol-2-yl)-5-(3 carboxymethxyphenyl)-2-(4sulfophenyl)-2H-tetrazolium (MTT) assay. Cell suspensions (2000 μl) containing 10000-15000 viable cells were plated in a 24 multiwell plate. After 24 hrs cells were treated with different concentrations of foretinib (0,01, 0,1, 0,5, 1, 2, 5 and 10 μM) for 72 hrs. Each experiment was done in triplicate. Results represent the median of the three experiments, each performed in triplicate.

### Protein expression analysis

Protein lysates containing equal amount of proteins, measured by a modified Bradford assay (BIORAD), were subjected to immunoprecipitation or direct Western blot.

Immunocomplexes were dectected with the enhanced chemiluminescence kit (ECL plus, Thermo Fisher Scientific (Rockford, IL). We used the following antibodies from Cell Signalling (Beverly, MA): anti-ERK, anti-phospho-pERK(T202/Y204), anti-AKT, anti-phospho-AKT (S473), anti-AXL, anti-phospho-AXL (Y702), anti-phospho-S6 ribosomal protein (S236/S253). Anti-α-tubulin was from Sigma (Sigma-Aldrich, St. Louis, MO). The following secondary antibodies from Bio-rad (Hercules, CA) were used: goat anti-rabbit IgG and rabbit anti-mouse IgG. Each experiment was done in triplicate. The Proteome Profiler Array has been performed by following manufacturer's protocol. (Human Phospho-Kinase Array Kit, R&D Systems). Briefly, SW620, SW480, LOVO and HCT116 cells were serum starved for 24 hrs and 300 μg of protein lysates were obtained and incubated over night with the human phospho-kinase array in order to analyse the phosphorylation profile of the 41 kinases.

### Microarray gene expression analysis of cancer cell lines

Agilent microarray analyses were performed to assess baseline gene expression profile for each cancer cell line using a one color labeling microarray system as described before [23]. Data were extracted from slide image using Agilent Feature Extraction software (v.10.5). The raw data and associated sample information were loaded and processed by Gene Spring® 11.5X (Agilent Technologies, CA, USA). For identification of genes significantly altered in resistant cells, total detected entities were filtered by signal intensity value (upper cut-off 100th and lower cut-off 20th percentile) and flag to remove very low signal entities. Data were analyzed using Student's *t* test (*p* < 0.05) with a Benjamani-Hochberg multiple test correction to minimize selection of false positives. Of the significantly differentially expressed RNA, only those with greater than 2-fold increase or 2-fold decrease as compared to the controls were used for further analysis. Subsequently, hierarchical clustering (condition tree) was applied to the data files. In this way, the relationships between the different groups are shown. The condition tree was displayed as a heat map, based on expression levels of the probe sets. Functional and network analyses of statistically significant gene expression changes were performed using Ingenuity Pathways Analysis (IPA) 8.0 (Ingenuity® Systems, http://www.ingenuity.com). Analysis considered all genes from the data set that met the 2-fold (*p*-value < 0.05) change cut-off and that were associated with biological functions in the Ingenuity Pathways Knowledge Base. The significance of the association between the data set and the canonical pathway was measured in 2 ways: 1- Ratio of the number of genes from the dataset that map to the pathway divided by the total number of genes that map to the canonical pathway is displayed; 2- Fisher's exact test was used to calculate a p value determining the probability that the association between the genes in the dataset and the canonical pathway is explained by chance alone.

### RNA interference

The small inhibitor duplex RNAs (siRNA) (ON-target plus SMARTpool) si-Human AXL (#L-003104-00) was from Dharmacon (Lafayette, CO). The siCONTROL Non-targeting Pool (#D-001206-13-05) was used as a negative (scrambled) control. Cells were transfected with 100 nmol/L siRNAs using Dharmafect reagent following manufacturer's instructions. The day before transfection, the cells were plated in 35 mm dishes at 40% of confluence in medium supplemented with 5% FBS without antibiotics. Cells were harvested at different time points (24, 48, 72 hrs) after transfection. Cell proliferation analysis or Western blot for AXL expression were performed.

### Generation of stable AXL shRNA

Five lentiviral constructs (pLKO.1puro) containing 21-mer short hairpin RNAs (shRNA) directed to various coding regions of *AXL* were kindly provided by Prof Rosamarina Melillo. LOVO cells were transfected with the pool of sh*AXL* or with non-targeting vectors (shCTR) by using Fugene reagent following manufacturer's protocol. Stable transfectants were selected in medium with 500ng/mL puromycin for 12 weeks and then amplified.

### Migration

Chambers of transwell (6.5 mm diameter, 8 μm pore size polycarbonate membrane, Corning) were used to evaluate the migratory capacity of shCTR cells and the shAXL transfected ones. A cell concentration of 50 x10^3^ cells in 200 μl medium without FBS was added to each migration (upper) chambers of transwell. Chemotaxis was induced by addition of 10% FBS to the medium the lower chamber. Cells were allowed to migrate from the upper compartment through the membrane towards the lower compartment along the chemo attractant gradient. After incubation for 24 hours, non-migrating cells were removed with cotton swabs, and the cells that migrated into the lower surface of the filters were stained with crystal violet.

### Growth curve

Sh-ctr cells, sh-axl pool cells and clone 1, clone 3, clone 5 and clone 8 were seeded with a concentration of 50 × 10 ^3^ at time 0 and harvested and counted after 24, 48, 72 and 96 hrs. Each day, cells in suspension were harvested and stained with 0,4% trypan blue for 5 min at room temperature. Unstained (viable) and stained (nonviable) cells were counted and the percentage of dead cells were reported.

### ELISA assay

CRC were grown to 70% of confluence and serum-starved for 24 hrs. GAS6 levels in culture supernatants were measured using a quantitative immunoassay ELISA kit (DuoSet ELISA Development Kit, R&D Systems). Samples were analysed at 450 nm with an ELISA reader (Model 550 microplate reader, Bio-Rad). Each experiment was performed in triplicate.

### Luminex

Cancer cell lines were grown to 70% of confluence and serum-starved for 24 hrs. TGF-β levels in supernatant were calculated using a Milliplex Map Kit (TGF-β single Plex 96-well Plate Assay Cat. #TGF-64K-019, Millipore). Plates were read on a Luminex 100 system.

### RNA extraction and reverse transcription polymerase chain reaction

For the validation of genes identified by gene expression profiling, quantitative real-time RT–PCR was performed on RNA isolated from CRC cells. Total RNA was collected from cultured cells using the RNeasy Kit (Qiagen, Crawley, West Sussex, UK) according to the manufacturer's instructions. Random-primed first strand cDNA was synthesized in a 50 μl reaction volume starting from 2 μg RNA by using the GeneAmp RNA PCR Core Kit ( AppliedBiosystems, Warringtone, UK). Quantitative Q-RT-PCR were performed by using SYBR Green PCR Master mix (Applied Byosystems) in the iCycler apparatus (Bio-Rad, Munich, Germany).

The quality of RNA was verified by electrophoresis through 1% agarose gel and visualized with ethidium bromide. Primers were designed by using a software available at http://bioinfo.ut.ee/primer3/ and synthetized by the CEINGE s.c.a.r.l. DNA synthetis facility (Naples, Italy). To exclude DNA contamination, each PCR reaction was performed on untrascribed RNA. Amplification reactions (25 μl final reaction volume) contained 200 nM of each primer, 3 mM MgCl2, 300 μM dNTPs, 1x SYBR Green PCR buffer, 0.1 U/μl AmpliTaq Gold DNA Polymerase, 0.1 U/μl AMP Erase, RNase-free water and 2 μl cDNA samples. Therma cycling conditions were optimized for each primer pair and are available upon request. To verify the absence of non-specific products, 80 cycles of melting (55 °C for 10 sec) were performed. In all cases, the melting curve confirmed that a single product was generated. Amplification was monitored by measuring the increase in fluorescence caused by the SYBR-Green binding to double-stranded DNA. Fluorescent threshold values were measured in triplicate and fold changes were calculated by the formula: 2- (sample 1 DCt – sample 2 DCt), where D*Ct* is the difference between the amplification fluorescent thresholds of the mRNA of interest and the b-actin mRNA.

### Cell proliferation ELISA Brdu

LOVO Cells and clones suspensions (100 μl) containing 5000 viable cells were plated in a 96 multiwell plate and serum starved after 24 hour. The day after BRDU assay was performed following manifacturer's instructions (Cell proliferation WlisaBrdu, Roche).

### Tumor xenografts in nude mice

Four- to six-week-old female balb/c athymic (nu1/nu1) mice were purchased from Charles River Laboratories. (Milan, Italy). The research protocol was approved and mice were maintained in accordance with the institutional guidelines of the Second University of Naples Animal Care and Use Committee. Mice were acclimatized at the Second University of Naples Medical School Animal Facility for 1 week before being injected with cancer cells and then caged in groups of 8.

All studies were conducted in accordance with the ‘Guide for the Care and Use of Laboratory Animals’ (NIH) and approved by the Institutional Animal Care and Used Committee (IACUC).

A total of 2 x10^6^ HCT116 cells re-suspended in 200 μl matrigel (BD Biosciences, Milan, IT): PBS (1:1) were implanted subcutaneously into the right flank of nude female mice. Orthotopic *in vivo* models. The orthotopic implantation was performed as described by Hoffman and colleagues (42). After one week mice were randomized and divided into two different treatment groups treated with vehicle or foretinib (n° 8 for each group). Group 1: vehicle (PBS/0,5% Tween 80) was administered through oral gavage every day, for 5 days a week. Group 2: foretinib (15 mg/kg) was prepared in vehicle 1 (0.5% Tween-80 in sterile PBS) and administered daily through oral gavage for 5 days a week. Mice were treated for three weeks and euthanized 1 week later. The body weights were monitored daily. Primary tumours in the cecum were excised and weighed. Orthotopic implantation: subcutaneous tumours derived from HCT116 cells were obtained as previously above. When the average tumours reached a mean volume of 500 mm^3^ animals were euthanized, the tumours were removed using sterile technique, divided into 2-3 mm-sized pieces, and harvested in PBS on ice. Mice were treated with antibiotics, ticarcillin (50 mg kg^−1^i.v.), two hours before and after tumour implantation. Animals were anesthetized with 2,2,2-tribomoethanol 97% TBE, Avertin (Sigma-Aldrich, St. Louis, MO, USA). TBE solution was prepared fresh daily by mixing 0.625 g of 97% crystalline TBE powder with 25 ml sterile 0.9% saline and then injected intraperitoneally at 0.01 ml/g body mass (250 mg/kg). The abdomen was prepped with betadine solution and the surgical site was isolated in a sterile fashion. A laparotomy of 0.5 cm was conducted; the cecum was exteriorized and isolated using pre-cut, sterile gauze. A warm saline solution was used to keep the cecum wet. Subsequently, the cecum wall was lightly damaged and a single tumour fragment from HCT-116 subcutaneous tumours was sutured to the mesenteric border of the cecum wall using 6.0 nylon surgical sutures. Upon completion, the cecum was replaced into the abdominal cavity and the abdominal wound was sutured using a 6.0 Ethicon absorbable stitches. (Ethicon Inc., Somerville, NJ). The final tumour was evaluated by caliper measurements using the following formula: π/6 x larger diameter x (smaller diameter)^2^. The presence of metastasis was evaluated in the peritoneum, liver, intestines, lungs, rectum and spleen and confirmed by histologic review. The tumour excised from each mouse was divided into 3 parts. One piece was formalin-fixed; the other two pieces were frozen at −80 C in RNAlater. Hematoxylin and eosin staining confirmed the presence of tumours in each sample. Student *t* test was used to evaluate the statistical significance of the results.

### Patients and samples

Histological blocks of 223 patients diagnosed with CRC, who had undergone surgical resection without any preoperative treatment between 2003 and 2011, were selected to obtain tissue microarrays upon informed consent. From the most representative areas of each donor tissue sample a single core with a diameter of 2 mm was arranged into one recipient paraffin block (3×2.5 cm) using a semiautomatic tissue arrayed (Galileo TMA). All cases were diagnosed at the National Cancer Institute Fondazione ‘G. Pascale’ of Naples and at Medical Oncology, Seconda Università degli Studi of Naples and staged according to the TNM classification (version dependent on year of diagnosis). Clinicopathological characteristics including demographics and staging features were evaluated.

### Immunohistochemistry (IHC)

Four μm-thick sections were deparaffinized and rehydrated and antigen retrieve technique was carried out in pH 6.0 buffer in a microwave for 3 minutes using standard histological technique. The primary antibodies were incubated overnight at 4°C at the concentration of 10ug/mL for anti-GAS6 (AF885, Goat mAb, R&D Systems) and 1:300 for anti-AXL (C89E7, Rabbit mAb, Cell Signaling), washed and then incubated with anti-Goat and anti-Rabbit secondary antibodies respectively. Appropriate positive and negative control were chosen. Evaluation has been done by two expert pathologists without prior knowledge of clinicopathological information (G. L; G.B). The scoring was performed considering percentage of tumour cells staining positively (positive and negative). In GAS6 positive samples, staining results were defined as low (1-19%) and high ( > = 20%). Whereas in AXL positive samples, staining expression levels were classified into low (1-19%) and high ( > = 20%) and intensity level was graded as low (1+), moderate (2+) or intense (3+). Both in AXL and GAS6 specimens 20% thresholds was chosen according with median scoring value obtained after statistical analysis. Furthermore, to better investigate AXL expression, a semiquantitative scale ranging from 0 to 300 based on histoscore (HSCORE) was obtained considering tumour cells staining percentage (0-100) multiplied by staining intensity (0-3).

### FISH analysis

Tissue array sections from paraffin-embedded tissue were heated for 4 h at 62°C and immediately deparaffinized in two rinses of 100% xylene for 10 min each. The slides were then treated with 0.3 M sodium chloride and 0.03 M sodium citrate for 20 min at 80°C, and with 0.05 mg/ml proteinase for 10 min at 37°C. AXL/CEN19q FISH Probe ( FG0088 ABNOVA) (10 ml) was applied to the tissue sections and covered with a coverslip. Both probe and target DNA were simultaneously denatured at 85°C for 5 min and incubated overnight at 37°C using the Hybrite System. Post-hybridization washes were performed according to the ‘rapid wash protocol’ provided by Vysis. Slides were counterstained with 406-diamidino-2-phenylindole 2HCl (DAPI). FISH was performed according to the manufacturer's instructions (Vysis). FISH data were collected using an Olympus BX 61 fluorescence microscope equipped with a cooled black-and-white camera controlled by the associated software (Olympus, Italy). Signals were evaluated by two independent evaluators (GL, GB) scoring at least 60 interphase nuclei in four different high power fields (HPF). The FISH results were scored as follow: specimens with the ratio AXL/CEN19 ≥ 2.0 were considered as amplified; polysomic were considered cases showing three or more CEP-19 signals per cell in more than 30% of the evaluated cells.

### Statistical analysis

The Student's *t* test was used to evaluate the statistical significance of differences between treatment effects of *in vivo* and *in vitro* data. Differences between categorical data were measured by χ*^2^* square test. Differences between continuous variables were investigated by the Mann-Whitney U test and Kruskal-Wallis test, when appropriate. Survival curves were plotted using the Kaplan-Meier method and compared using the Log-rank test. All the tests were two-sided, with p value of < 0.05 considered to indicate statistical significance. All statistical analyses were carried out using the SPSS package (version 21.0 for Windows, SPSS Inc., USA).
